# Supplementary material for: Plant Histone HTB (H2B) Variants in Regulating Chromatin Structure and Function
Source: Plants (Basel). 2020 Oct 25;9(11):1435. doi: 10.3390/plants9111435 (PMC7694166; doi:10.3390/plants9111435)
Supplement: Supplementary file 1 [file plants-09-01435-s001.pdf]

Supplementary Materials

Plant histone HTB (H2B) variants in regulating chromatin structure and function

Janardan Khadka, Anat Pesok, Gideon Grafi*

French Associates Institute for Agriculture and Biotechnology of Drylands, Jacob Blaustein Institutes for Desert Research, Ben-Gurion University of the Negev, Midreshet Ben Gurion 84990, Israel.

*Correspondence: ggrafi@bgu.ac.il

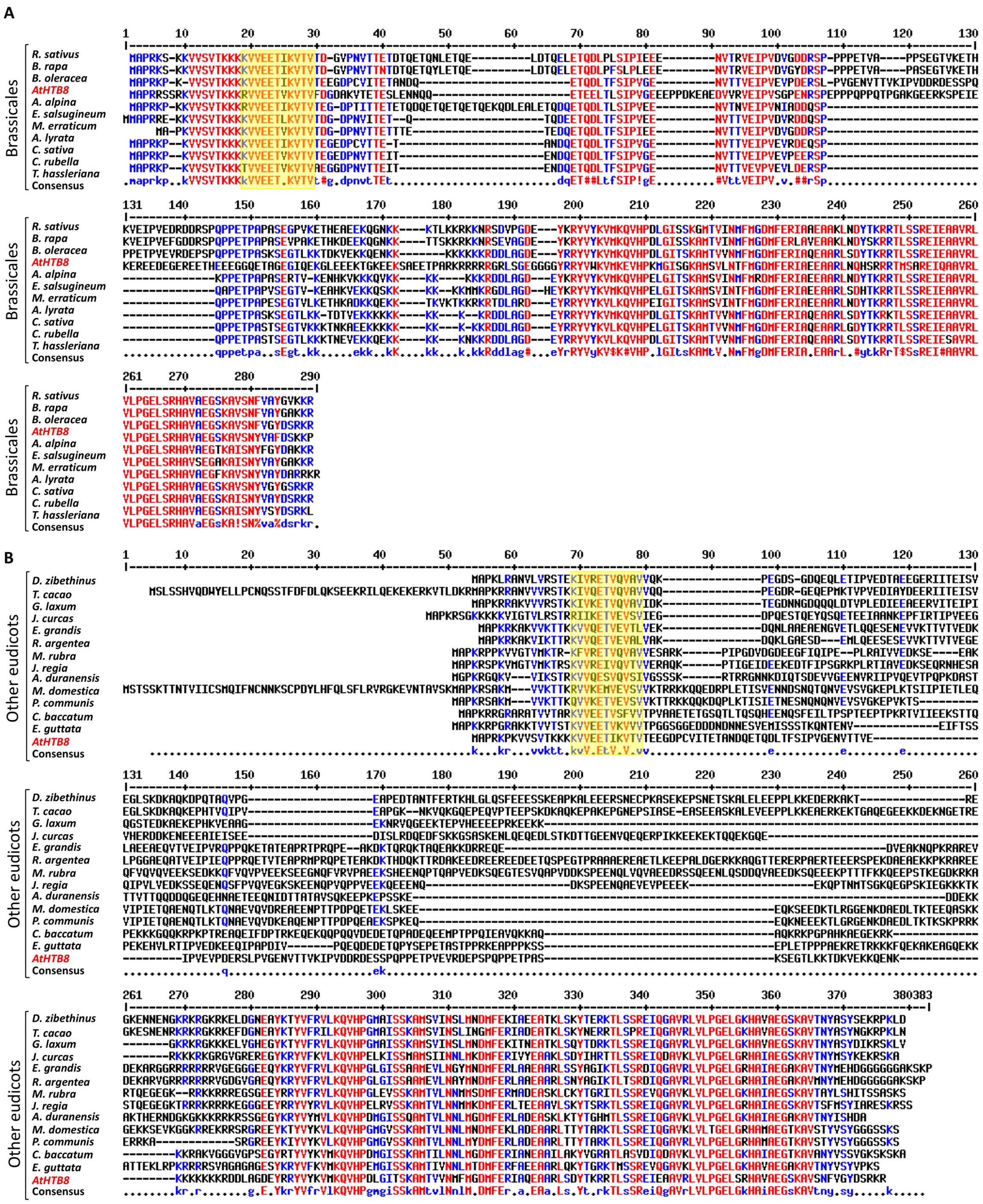

Fig. S1. Amino acid sequence alignments of HTB8-like proteins (Multalin interface; Corpet, 1988). (A) Alignments of HTB8-like proteins in Brassicales. (B) Alignments of HTB8-like proteins in other eudicot species. Note that all HTB8-like proteins share high amino acid sequence similarity of the C-terminal region. The yellow box marks the conserved domain in the N-terminal extension of HTB8 orthologs. Species abbreviations: *C. baccatum, Capsicum; E. guttata, Erythranthe; T. hassleriana, Tarenaya; M. erraticum, Microthlaspi; A. alpine, Arabis; E. salsugineum, Eutrema; R. sativus, Raphanus; B. rapa, Brassica; B. oleracea, Brassica; C. sativa, Camelina; C. rubella, Capsella; A. lyrata, Arabidopsis; A. thaliana, Arabidopsis; J. curcas, Jatropha; G. laxum, Gossypium; T. cacao, Theobroma; D. zibethinus, Durio; A. duranensis, Arachis; J. regia, Juglans; M. rubra, Morella; M. domestica, Malus; P. communis, Pyrus; E. grandis, Eucalyptus; R. argentea, Rhodamnia.*

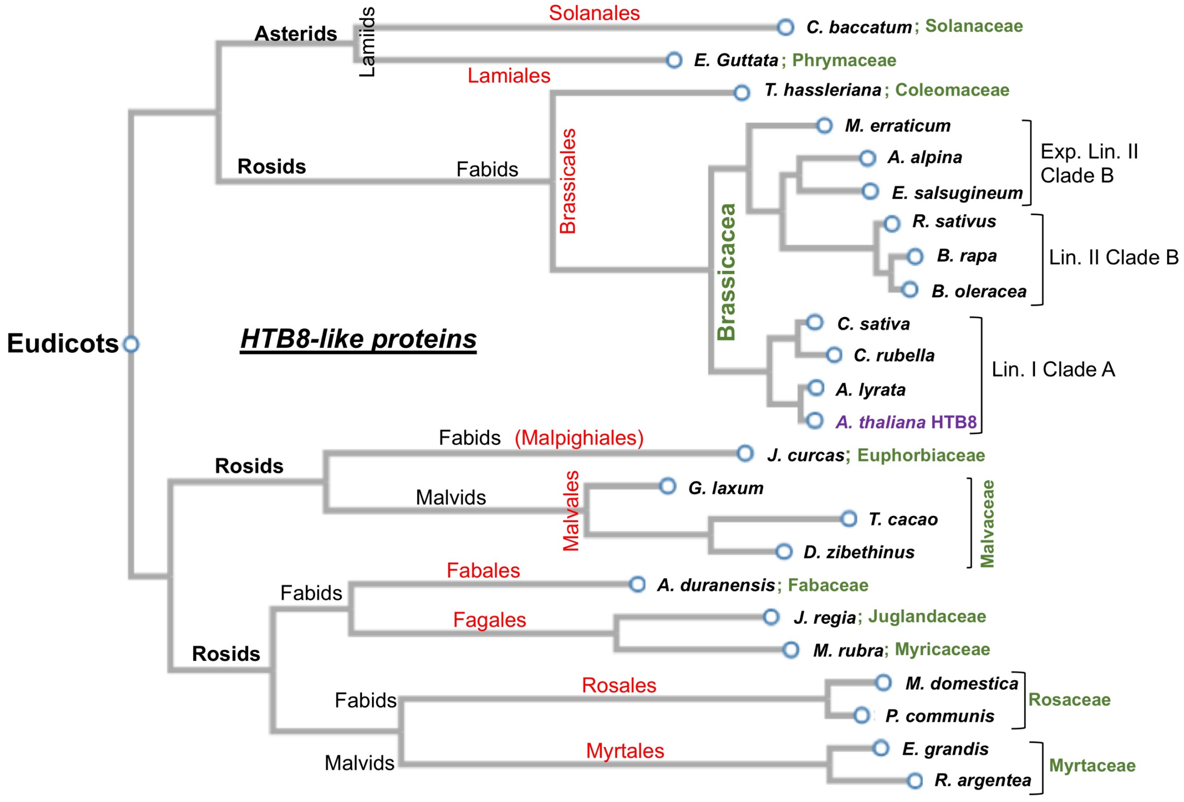

Fig. S2. A phylogenetic tree of HTB8-like proteins within the Eudicot clade of angiosperms. Species within the Rosids clade distributed between Fabids and Malvids and the Asterids clade (Lamiids) are presented. Orders are in red and families are colored green. The Brassicaceae species are clustered according to their phylogeny (Gou et al., 2017) including expanded lineage II (Exp. Lin. II), clade B; Lin II, clade B and Lin I, clade A. Species abbreviation as in Fig. S1.

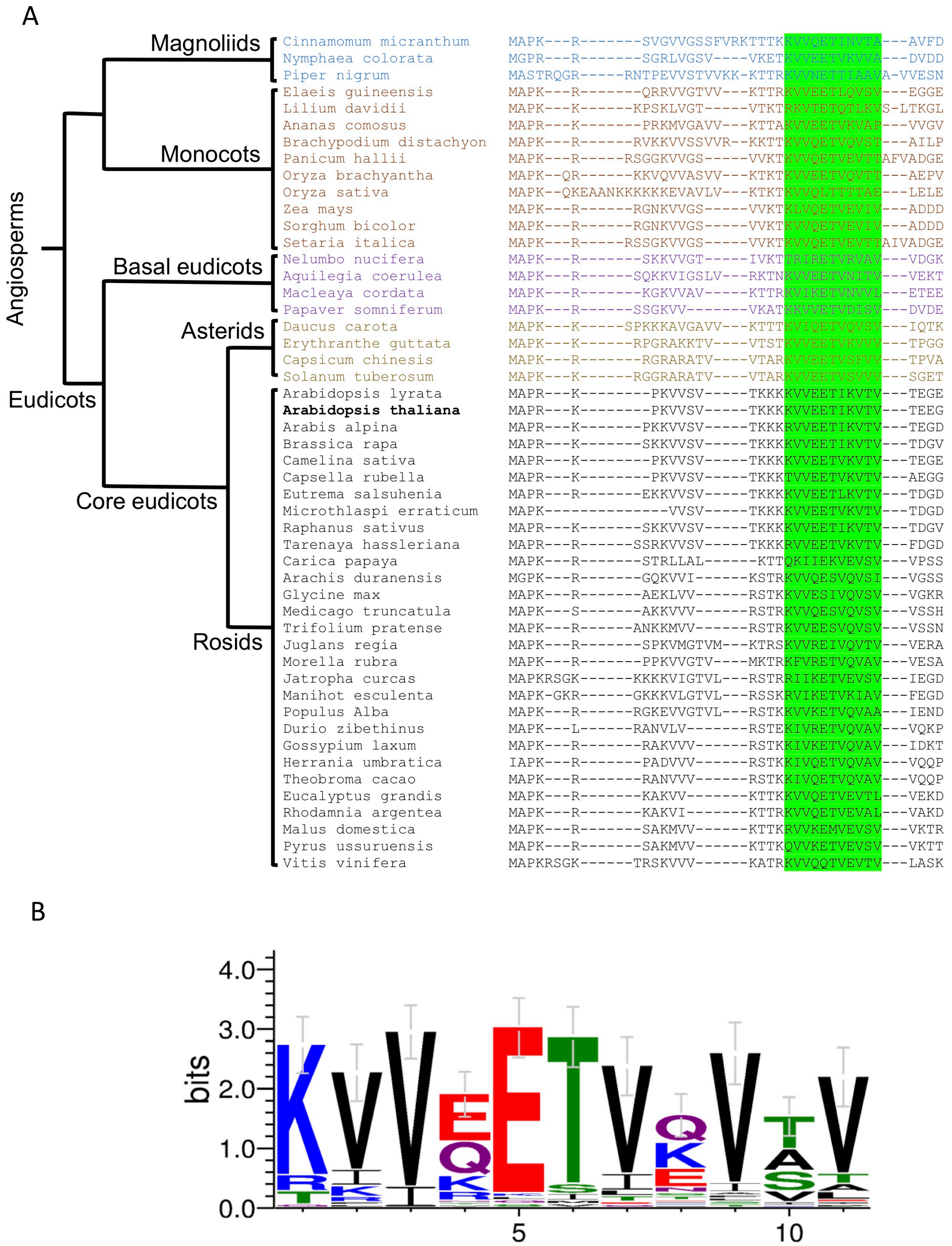

Fig. S3. A conserved motif in the N-terminal extension of HTB8 orthologs in angiosperms. (A) Alignment of N-terminal sequences of representative HTB8 orthologs from various species of angiosperm clades. The conserved motif is highlighted in green. (B) Motif logo generated using WebLogo (http://weblogo.berkeley.edu/) showing the consensus core sequence KVVxETVxVxV of the conserved motif.

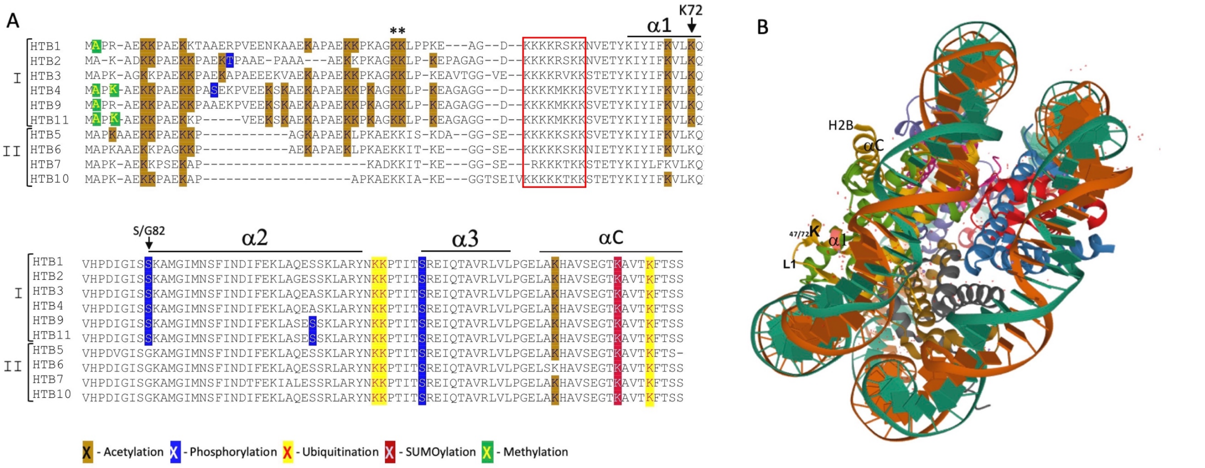

Fig. S4. (A) Posttranslational modifications of *Arabidopsis* HTB variants based on the Plant PTM viewer (https://www.psb.ugent.be/webtools/ptm-viewer/protein.php) and Bergmüller et al. [30]. The histone fold helices α1-α3 and the C terminal (αC) helices are indicated. PTMs are highlighted as follows: brown, acetylation; green, methylation; blue, phosphorylation; red, SUMOylation and yellow, ubiquitination. Note, K39/K40 (asterisks) and K72 were found to be acetylated in class I HTBs but not in class II. The S to G change at position 82 is indicated. Red box indicates the putative nucleolar localization/retention signal. (B) Nucleosomal disc-like structure modeling the histone fold of the human H2B type 1-J (colored gold) with the α1 and αC helices facing outside the nucleosomal disc. The merged position of K47 (humanH2B type 1-J) and K72 (HTB9) is indicated ($_{72/47}$K).

Protein sequences analyzed in this study

Arabidopsis thaliana

>HTB1/At1g07790
MAPRAEKKPAEKKTAAERPVEENKAAEKAPAEKKPKAGKKLPPKEAGDKKKKRSKKNVETYKIYIFKVLKQVHPDIGISSKAMGIMNSFINDI
FEKLAQESSKLARYNKKPTITSREIQTAVRLVLPGELAKHAVSEGTKAVTKFTSS
>HTB2/At5g22880
MAKADKKPAEKKPAEKTPAAEPAAAAEKKPKAGKKLPKEPAGAGDKKKKRSKKNVETYKI
YIFKVLKQVHPDIGISSKAMGIMNSFINDIFEKLAGESSKLARYNKKPTITSREIQTAVRLVLPGELAKHAVSEGTKAVTKFTSS
>HTB3/At2g28720
MAPKAGKKPAEKKPAEKAPAEEEKVAEKAPAEKKPKAGKKLPKEAVTGGVEKKKKRVKKS
TETYKIYIFKVLKQVHPDIGISSKAMGIMNSFINDIFEKLAQEASKLARYNKKPTITSREIQTAVRLVLPGELAKHAVSEGTKAVTKFTSS
>HTB4/At5g59910
MAPKAEKKPAEKKPASEKPVEEKSKAEKAPAEKKPKAGKKLPKEAGAGGDKKKKMKKKSVETYKIYIFKVLKQVHPDIGISSKAMGIMNSFIN
DIFEKLAQEASKLARYNKKPTITSREIQTAVRLVLPGELAKHAVSEGTKAVTKFTSS
>HTB5/At2g37470
MAPKAAEKKPAEKKPAGKAPAEKLPKAEKKISKDAGGSEKKKKKSKKSVETYKIYIFKVLKQVHPDVGISGKAMGIMNSFINDIFEKLAQESS
KLARYNKKPTITSREIQTAVRLVLPGELAKHAVSEGTKAVTKFTS
>HTB6/At3g53650
MAPKAAEKKPAGKKPAEKAPAEKLPKAEKKITKEGGSEKKKKKSKKNIETYKIYIFKVLKQVHPDIGISGKAMGIMNSFINDIFEKLAQESSR
LARYNKKPTITSREIQTAVRLVLPGELSKHAVSEGTKAVTKFTSS
>HTB7/At3g09480
MAPKAEKKPSEKAPKADKKITKEGGSERKKKTKKSTETYKIYLFKVLKQVHPDIGISGKAMGIMNSFINDTFEKIALESSRLARYNKKPTITS
REIQTAVRLVLPGELAKHAVSEGTKAVTKFTSS
>HTB8/At1g08170
MAPRKPKVVSVTKKKKVVEETIKVTVTEEGDPCVITETANDQETQDLTFSIPVGENVTTVEIPVEVPDERSLPVGENVTTVKIPVDDRDESSP
QPPETPVEVRDEPSPQPPETPASKSEGTLKKTDKVEKKQENKKKKKKKKRDDLAGDEYRRYVYKVMKQVHPDLGITSKAMTVVNMFMGDMFER
IAQEAARLSDYTKRRTLSSREIEAAVRLVLPGELSRHAVAEGSKAVSNFVGYDSRKR
>HTB9/At3g45980
MAPRAEKKPAEKKPAAEKPVEEKSKAEKAPAEKKPKAGKKLPKEAGAGGDKKKKMKKKSVETYKIYIFKVLKQVHPDIGISSKAMGIMNSFIN
DIFEKLASESSKLARYNKKPTITSREIQTAVRLVLPGELAKHAVSEGTKAVTKFTSS
>HTB10/At5g02570
MAPKAEKKPAEKAPAPKAEKKIAKEGGTSEIVKKKKKTKKSTETYKIYIFKVLKQVHPDIGISGKAMGIMNSFINDIFEKLAQESSRLARYNK
KPTITSREIQTAVRLVLPGELAKHAVSEGTKAVTKFTSS
>HTB11/At3g46030
MAPKAEKKPAEKKPVEEKSKAEKAPAEKKPKAGKKLPKEAGAGGDKKKKMKKKSVETYKIYIFKVLKQVHPDIGISSKAMGIMNSFINDIFEK
LASESSKLARYNKKPTITSREIQTAVRLVLPGELAKHAVSEGTKAVTKFTSS

HTB8 orthologs

Magnoliids

>Cinnamomum micranthum - RWR72246.1 late histone H2B.2.2-like protein; **Lauraceae**
MAPKRSVGVVGSSFVRKTTTKKVVQETINVTAAVFDPNNTEEEVEELNESLPTPTTKKVKVVEGPSQTSVTTPQEDENDTKESTPHPQEEEND
TTESTPHPQEEKNDTTESIPPQVENPPPPTKTTIEKKLNKEVGKEKEKEKEKKRRKKRRKVGVEMGYKRYVFMVLKQVHPGMGISSNAMTVLN
GLMNDMFEKLAAEASKLCLYTKRATMTSREIQGAVRLVLPGELGKHAIAEGTKAVANYMTNDRATSS
28.1 kDa

>Nymphaea colorata - XP_031483632.1 histone H2B-like; **Nymphaeaceae**
MGPRRSGRLVGSVVKETKVVEETVKVVADVDDLSSLEPSAISAGIIREIPVVEIKESKTPQKKVEPEAAPFPLEKKQKGIEAKEVEEKEQKGH
VDQKKERKPQELNRRRKRLRRNEEGGVGAYRSYVYKVLKQVHPELGISSKAMDVLNGFMGDMFERLAEEAATLQKHTGRRTLSSREIQMAVRL
VLPGELGKHAISEGSKAITNYYRYPEKKRR
24.45akDA
>Piper nigrum; **Piperaceae**
MASTRQGRRNTPEVVSTVVKKKTTRKVVNETTIAAVAVVESNEPPIVKTVPVEEESSDSVINVEAGKTPPKEVPIARPRSDAAKGTNQRKDGA
ATIAETQQPPEKLREEKHAMEEVKKGSKRRKGERKEGEKKRKRRKKRWSYNEEMSGYSRYVFRVLKQVHPDLAISSKAMAVLNAFVWDMFERL
AGEAGKLADYTRRATLSSREIQDAVRLVLPGELGKHAISEGSKAVTNYVTNDD
26.7 kDa
Monocots:

>Elaeis guineensis - XP_010940500.1 histone H2B.11; **Arecaceae**
MAPKRQRRVVGTVVKTTRKVVEETLQVSVEGGEGNGDQNDSKESKVVEVEVEVEEKKAQESQTSVTHLEDSQAKEKAEEEKTNKRNKKSKQAP
KERAEEPPQRDKKQEKDGRREGKDQEEPPVGEGKQKGGGVVVEGEEKGKKRGRGGKRRRRKRYGGGVEGIGGGYKRYVFRVLKQVHPGMAVSS
RAMAVLDGMMIDMFERLADEAARLSQYAGKATLSSREIQDAVRLVLPGELGKHAISEGTKAVSKYMSADHGT
28.47 kDa
>Lilium davidii- mgH2B.in; **Liliaceae**

MAPKKKPSKLVGTVTKTRKVTETQTLKVSLTKGLKPEDQQTTTNKFEVSVTGKQSKTQPLIVSTNTNLVPKKEKEESPTTTLMVKKKRKNRKA
GGEYKRYVYMVLKTVHPDMTVSSKAMMVMEGMMQDMFERLVTEAVRLVQYMKKATLTCREIQTAVMLVLPGELGKHAVSEGAKAITNYMAAVG
SGNGGAA/
21.25 kDa
>Ananas comosus - XP_020113371.1 histone H2B.3-like; **Bromeliaceae**
MAPRKPRKMVGAVVKTTAKVVEETVKVAPVVGVGDGDGDGDGAEEVEEAAVPLKDSKVVQVVVVGGEKGDGEVPEANDGRDEPEKRKEAMEVD
ENQAPKETGEESKRRGRGRPLKERGPETPTEKSEIPPANKKKEKDRGGRSEGEAEEGKGRRRRRRKRRFGSAGDAGSGGVGGYKRYVFRVLKQ
VHPELGASARAMQVLDMMMADMFQRLAEEAARLSKYTGRATLTSREIQNAVRLVLPGELGRHAVSEGTKAVTNYMASQSS
28.8 kDa
>Brachypodium distachyon; **Poaceae**
MAPKRRVKKVVSSVVRKKTTKVVQETVQVSTAILPDDSAQPEPEVVVDVSTPATVVKHVEVTSDVGDDQATAAGATAVNNKPPQSKSPDKPAD
DNQATAPAVPSLQSQETQTQDPNEKKKTPQQEIVVVTTKGPPGLEPEEKTKKLQPDAPETPKQAGGAGTGGKDEAAAPKKKKKKKKKKRKARR
GGGRRRGPVGDMGMMGGGYKRYVYRVLKQVHPDMGASGRAMEVLDMMMGDMFERLADEAARLAKVAGRATLSSREVQNAVRLVLPGELAKHAI
SEGTKAVTSYMSLA
31.27 kDa
>Oryza brachyantha - XP_006661574.1 PREDICTED: late histone H2B.L4-like; **Poaceae**
MAPKQRKKVQVVASVVKTKTKVVEETVQVTTAEPVSGSTVQVEVETDAKKTTAVQSQEREEPNAQGRRKGGGGGGGRRRRSRRGGAGAGGGYK
RYVWRVLKQVHPDMGASGRTMDVLDTMMSDMFERLAEEASRLSKVSGRATLTSREVQSAVRLVLPGDLANHAISEGTKAVSNYLS
19.15 kDa
>Oryza sativa - XP_015612586.1 late histone H2B.L4; **Poaceae**
MAPKQKEAANKKKKKKEVAVLVKTKTKVVQLTTTTAELELEPTVTVQVDDNKTGAAADETPPVVPLQSQETQDPNEPKAAAAKKKKRAGHGRK
RSRRRRGGALEYGGYKRYVWRVLKQVHPDLGASAQTMDVLDMMMADMFERLADEASRLSKLSGRLTLTSREVQSAVRLVLPADLANHAISEGT
KAISNYLS
21.34 kDa
>Panicum hallii - XP_025799366.1 histone H2B.9-like; **Poaceae**
MAPKRRSGGKVVGSVVKTKVVQETVEVTTAFVADGEPGQRATEDLALAPPAVDASGGSRSRVVHIEQATSKRGRGGRREEEKPAPPAEEAAQE
PPVAQSQETQDPNEEQEEEEDAGKKKKKKPPQQELQDEEPPETPRVASERKTAAAKRTPQQQQKRGGGGAGGGDKTKTTKAKKGGRRRLGQA
SPGGDAGMGGVGGYKRYVWRVLKQVHPELGVSGNAMRVLDMMMADMFERLADEAARLSKVSGRATLSSREVQSAVRLVLPGELSRHAMSEGTK
AISKYMSYDA
30.97 kDa
>Setaria italica; **Poaceae**
MAPKRRSSGKVVGSVVKTKVVQETVEVTTAIVADGEPEQQLAPGALALAPRTGEVSRSKVVHVEITTPDSDNTTGRSSAKQQPTAKRGRGGRR
EEEKPPAPAEEAAQEPPPQSLETQEPNEEEEEEDVDVSKKRRKPPPQQRRRDEEEPETPRVASERKTAGTKTTPQKQKKRGGGGGGGGKAKTG
RRRRLGEASPGGDAGMGGMGGYKRYVWRVLKQVHPELGVSGHAMRVLDMMMADMFERLADEAARLSKAAGRATLSSREVQSAVRLVLPGELGR
HAMSEGTKAISKYMSYDDA
32.16 kDa
>Sorghum bicolor - XP_002462010.1 histone H2B.4; **Poaceae**
MAPKRRGNKVVGSVVKTKVVQETVEVIVADDDDTAEAEQQMVPEALAVAPSAVDVSGSTVVHVVEVTTPDGGDNATGSNVKQPAVAKRGRGRR
EEEKEKQPAPPEDSVLVPQSQETQDPNEEEEDQEDASKKKKQKQKQRQQDEDDEAQPETPRVASERKKATPKKAKAKAQPQQQAGGGGDAGKK
RPKARRRLGQASAGGDAGMGGVGGYKRYVWRVLKQVHPDLGVSGHAMQVLDMMMADMFERLADEAARLSKATGRMTLTSREVQSAVRLVLPGD
LGKHAIAEGTKAISKYMSYA
32.19 kDa
>Zea mays - XP_008670031.1 histone H2B.5; **Poaceae**
MAPKRRGNKVVGSVVKTKLVQETVEVIVADDDGLHAEKQQVPEALALAHPTVDVSGSTVVHVVEVTAKRGRGGGGGGGGGGRRNEGKPPPEED
SAAVPVPQSQETQDPNEELEFELEDEEEKQPETPRVASEKRKKAATPTKKTKTQQPRRRRQRLGQASSGGDAGMGGVGGYRRYVWRVLKQVHP
DLGVSGHAMQVLDMMMADMFERLAEEAARLSKATGRATLTSREVQSAVRLVLPGELGRHAISEGTKAISKYMSYAA
28.17 kDa

Dicots:

Basal eudicots

>Nelumbo nucifera - XP_010267330.1 late histone H2B.2.2-like; **Nelumbonaceae**
MAPKRSKKVVGTIVKTTRIRETVRVAVVDGKKAEGETEKETIVESTKEPIRVAIEDVRAPEDQLVQKEPRRVMVDDKVQEEDG
AVAVSPETRERGQQEKINQEGKEEKKTEQKGEDKKKKKRERRKGGRRRKEGGEEYKRYVFRVLKQVHPGMGISSKAMAVLNGF
MNDMFERLAGEATRLSQYTGKMTLSSREIQGAVRLVLPGELGKHAIAEGTKAVTNYMSNKTAAAQAA

>Aquilegia coerulea - PIA54901.1; **Papaveraceae**
MAPKRSQKKVIGSLVRKTNKVVEETVNITVVEKTKGAEAGNKETEQEETAISTKDPVSTPGEKQQKEQPKKGVGKQLKLEAPT
QKKEETKSKKNEKSTTTTTTTTSSAKEGEEKKGKKSGRKRMLDTGETYNTYVYKVLKQVHPDLGITFKGMMVLNGFMNDMFER
LAREASKLTDYTGKKTMSAREIQGAVRLVLPGELGKHAIVEGTKAITTYFSNSS

>Macleaya cordata - OVA17917.1 Histone H2B; **Papaveraceae**
MAPKRKGKVVAVKTTRKVIKETVNVVLETEEPLQAEEENEEETDEIAVSTKEPVKVVVAVKDKTTEEDEDQSVHDQKKKKNQE
EPKKATSSLNLEGPQKKQQQLQDEEKKTAQDGGGEEMEKQGPNNQNKEENKTTTQDGVEDKEKKKKPGRKGSGRKRNKEGGEG
YKRYVFKVLKQVHPGMSISSKAMTVLNGLMNDMFERLADEAARLSKYTGRMTLSSREIQSAVRLVLPGELGKHAIAEGIKAVT
TYMSEDTKLGSKS

>Papaver somniferum - XP_026418705.1 histone H2B-like; **Papaveraceae**

MAPKRSSGKVVVKATKKVVETVDISVDVDEIGEVTNPEETITKDINVVVEETIKKKPQQETAAKVTPLKIEGAKDQTEKKKDG
KEEAKKTQDGEETEKSQEENEKTKVEKKKVKKTQEKGKEEKEKKQRTGRKRKMFTGIEGEYKRYVFKVLKQVHPDLGVSSRAM
VIINGLMNDMFERLAKEAAKLCDYTGRMTMSSREIQAAVRLVLPGELGKHAISEGVKAVTNYTSHE

Core eudicots; Asterids

Apiales

>Daucus carota - XP_017234272.1 PREDICTED: late histone H2B.L4-like; **Apiaceae**
MAPKKSPKKKAVGAVVKTTTKVIQETVQVSVIQTKPKPQQETPQTENNKNGPKDIEIQDVTTPTPTPKKATKTIPTQDTAKKT
KKDSAQGATKKRKRSVEGYKRYVYKVLKQVHPDIGISSKAMTIVNNLMTDMFERLADEAARLTKYTKKMTLSSREIQGAVKLV
LPGELGKHAVAEGAKAVTNYVQYASGPSKP

Lamiales

>Erythranthe guttata - XP_012838320.1 PREDICTED: probable histone H2B.3; **Phrymaceae**
MAPKKRPGRAKKTVVTSTKVVEETVKVVVTPGGSGGEDDDNDNNESVEMISSSTKQNTENVEIFTSSPEKEHVLRTIPVEDKE
EQIPAPDIVPQEQDEDETQPYSEPETASTPPRKEAPPPKSSEPLETPPPAEKRETRKKKFQEKAKEAGQEKKATTEKLRPKRR
RRSVAGAGAGESYKRYVFKVMKQVHPEMGISSKAMTIVNNLMTDMFERFAEEAARLQKYTGRKTMSSREVQGAVKLVLPGELG
KHAVAEGAKAVTNYVSYVPKS

Solanales

>Capsicum chinense - PHU15803.1 Histone H2B type 1-M; **Solanaceae**
MAPKRRGRARATVVTARKVVEETVSFVVTPVAAETETGSQTLTQSQHEENQSFEILTPSPTEEPTPKRTVIIEEKSTTQPEKK
KGQQKRPKPTREAQEIFDPTRKEQEKQQPQQVDEDETQPADEQEEMPTPPQIEAVQKKAQAQKRKPGPAHKAEGEKRKKKRAK
VGGGVGPSEGYRTYVYKVMKQVHPDMGISSKAMTILNNLMGDMFERIANEAAILAKYVGRATLASVDIQDAVKLVLPGELGKH
AIAEGTKAVANYVSSVGKSKSKA

>Solanum tuberosum **-** XP_006347209.1 PREDICTED: histone H2B.3-like; **Solanaceae**
MAPKKRGGRARATVVTARKVVEETVSVVVSGETETESQTLTEENQSFEILTPLPYEEPTPKRTINVQDKSEGKKAQQRKPDPA
QQVDEVETQPADEPEEMPSPPKKEAVRKKAQKRKPDPAQRVDEDETQPAEEPEEMPTPPKMEADQKKAQKAKGGGGERKKKRA
KVGGGVGPSEGYRRYVFRVMKQVHPDMGISSKAMMILNNLMGDMFERIANEAAILTKYAGRATLASVDIQDAVKLVLPGELGK
HAIAEGTKAVANYVTSVEKSKSKP

Core eudicots; rosids

Brassicales

>Arabidopsis lyrata - XP_002892444.1 histone H2B.2; **Brassicaceae**
MAPRKPKVVSVTKKKKVVEETIKVTVTEGEDPCVTTETANDQETQDLTFSIPVGENVTTVEIPVEVRDEQSPQPPETPASKSE
GTLKKTDTVEKKKKKKKKKRDDLAGDEYRRYVYKVMKQVHPDLGITSKAMTVVNMFMGDMFERIAQEAARLSDYTKRKTLSS
REIEAAVRLVLPGELSRHAVAEGSKAVSNYVGYGSRKR

>Arabidopsis thaliana - NP_172295.1 HTB8; **Brassicaceae**
MAPRKPKVVSVTKKKKVVEETIKVTVTEEGDPCVITETANDQETQDLTFSIPVGENVTTVEIPVEVPDERSLPVGENVTTVKI
PVDDRDESSPQPPETPVEVRDEPSPQPPETPASKSEGTLKKTDKVEKKQENKKKKKKKKRDDLAGDEYRRYVYKVMKQVHPDL
GITSKAMTVVNMFMGDMFERIAQEAARLSDYTKRRTLSSREIEAAVRLVLPGELSRHAVAEGSKAVSNFVGYDSRKR

>Arabis alpina - KFK43131.1; **Brassicaceae**
MAPRKPKKVVSVTKKKRVVEETIKVTVTEGDPTITTETETQDQETQETQETQEKQDLEALETQDQETQDLTLSIPVEENVTNV
EIPVNIADDQSPKPPETPAPASERTVKENHKVKKKQVKKKKKKKRDDLAGDEYKRYVYKVMKQVHPELGITSKAMSVINMFMR
DMFERIAEEAARLNDYTKRRTLSSREIEAAVRLVLPGELSRHAVAEGTKAISNYFGYDAKKR

>Brassica rapa - XP_009148076.1 histone H2B.2; **Brassicaceae**
MAPRKSKKVVSVTKKKKVVEETIKVTVTDGVPNVTTNTDTQETQYLETQELDTQELETQDLPFSLPLEEENVTRVEIPVDVGY
DRSPPPPETVAPASEGTVKETHKVEIPVEFGDDRSPQPPETPAPASEVPAKETHKDEEKQGNKKTTSKKRKKNRSEVAGDEYK
RYVYKVMKQVHPDLGISSKAMTVINMFMGDMFERLAVEAAKLNDYSKRRTLSSREIEAAVRLVLPGELSRHAVAEGSKAVSNF
VAYGAKKR

>Camelina sativa - XP_010475607.1 PREDICTED: histone H2B.2-like; **Brassicaceae**

MAPRKPKVVSVTKKKKVVEETVKVTVTEGEDPNVTTEITENDQETQDLTFSIPVGENVTTVEIPVEVPEERSPQPPETPASTS
EGTVKKKTNKAEEKKKKKKKKKKRDDLAGDEYRRYVYKVLKQVHPELGITSKAMTVVNMFMGDMFERIAQEAARLGDYTKRRT
LSSREIEAAVRLVLPGELSRHAVAEGSKAISNYVAYDSRKR

>Capsella rubella - XP_006306103.1 histone H2B.2; **Brassicaceae**
MAPRKPKVVSVTKKKTVVEETVKVTVAEGGDPNVTTEITENDQETQDLTFSIPVGENVTTVEVPVEVLDERSPQPPETPASTS
EGTLKKKTNEVEKKQEKKKKKNKKRDDLAGDEYRRYVYKVLKQVHPDLGITSKAMTVVNMFMGDMFERIAQEAARLSDYTKRR
TLSSREIESAVRLVLPGELSRHAVAEGSKAISNYVSYDSRKL

>Eutrema salsugineum - XP_006417719.1 histone H2B.2; **Brassicaceae**
MMAPRREKKVVSVTKKKKVVEETLKVTVTDGDPNVITETQTQDEETQDLTFSIPVEENVTTVEIPVDVRDDQSPQAPETPAPV
SEGTVKEAHKVEKKQSKKKKKKMMKRGDLAGDHEYKRYVYKVMKQVHPELGITSKAMSVINTFMGDMFERIAEEAARLSDHTK
RRTLSSREIEAAVRLVLPGELSRHAVSEGAKAISNYVAYGAKKR

>Microthlaspi erraticum - CAA7033736.1; **Brassicaceae**
MAPKVVSVTKKKKVVEETVKVTVTDGDPNVTTETTTETEDQETQDLTFSIPIEENVTTVEIPVDVGDDRSPQPPETPAPESEG
TVLKETHKADKKQEKKTKVKTKKRKRTDLARDEYRRYVYKVLKQVHPEIGITSKAMSVINTFMGDMFERIAEEAARLNDYTKR
RTLSSREIEAAVRLVLPGELSRHAVAEGFKAVSNYVAYDARRKR

>Raphanus sativus - XP_018484528.1 histone H2B.2; **Brassicaceae**
MAPRKSKKVVSVTKKKKVVEETIKVTVTDGVPNVTTETDTQETQNLETQELDTQELETQDLPLSIPIEEENVTRVEIPVDVGD
DRSPPPPETVAPPSEGTVKETHKVEIPVEDRDDRSPQPPETPAPASEGPVKETHEAEEKQGNKKKTLKKRKKNRSDVPGDEYK
RYVYKVMKQVHPDLGISSKGMTVINMFMGDMFERIAAEAAKLNDYTKRRTLSSREIEAAVRLVLPGELSRHAVAEGSKAVSNF
VAYGVKKR

>Tarenaya hassleriana - XP_010557373.1 PREDICTED: histone H2B.2; **Cleomaceae**
MAPRRSSRKVVSVTKKKRVVEETVKVTVFDGDAKVTETESLENNQQETEELTLSIPVGEEEPPDKEAEDVVRVEIPVSGPENR
SPEPPPQPPQTPGAKGEERKSPEIEKEREEDEGEREETHEEEGGQETAGEGIQEKGLEEEKTGKEEKSAEETPARKRRRRRGR
LSGEGGGGYRRYVWKVMKEVHPKMGISGKAMSVLNTFMGDMFERIAEEAARLNQHSRRRTMSAREIQAAVRLVLPGELSRHAV
AEGSKAVSNYVAFDSKKP

>Carica papaya - XP_021901556.1 histone H2B.8-like; **Caricaceae**
MAPKRSTRLLALKTTQKIIEKVEVSVVPSSGREQEITDVAVQKSPVKVIPVEEKSRKTVRIPVEETPSLKTIPVKTPEKEQET
IDDQEPVTTSEEVAADNEQEQEKEEETDQTQEGITSSEPAGTTKEEKVEKRPSRRGRPRRRRKKKKGSDEGNYKGYKRYVFKV
LKQVHPELAISSKAMVIINGFMNDMFERLADEAANLSRYSHKATLSSKEIQGAVRLVLPGELSKHATAEGSKAVTNYMSFPLH
NS

Fabales

>Arachis duranensis - XP_015935854.1 histone H2B; **Fabaceae**
MGPKRGQKVVIKSTRKVVQESVQVSIVGSSSKRTRRGNNKDIQTSDEVVGEENVRIIPVQEVTPQPKDASTTTVTTQQDDQGE
QEHNAETEEQNIDTTATAVSQKEEPKEPSSKEDDEKKAKTHERNDGKGKKKRRKRSSGEGYKRYVYMVLKQVHPDMGISSQAM
TVLNNLMGDMFERLADEASKLKTYTGHMTLSSREIQGAVKLVLPGELGKHAIAEGAKAVTNYISHDA

>Glycine max - KRH30545.1 hypothetical protein GLYMA_11G191600; **Fabaceae**
MAPKRAEKLVVRSTKKVVESIVQVSVVGKRLTQVIPQAQKVSPNSDITTENKAEQENNTHQDGGVQNQEEEQKGVVNEEAKEE
KNKSKTAKEQNGKEKKRGRKKRNIEGYQRYVYGVLKQVHPEMGISSKCMTALNNLMNDMFERLTFEVSKLTDYTGHMTLSSRE
IQGVVRLVLPGELEKHAIAEGVKAVNNYTSYDA

>Medicago truncatula - Medtr2g084480.1 K11252 histone H2B (H2B); **Fabaceae**
MAPKSAKKVVVRSTRKVVQESVQVSVVSSHKRSTRGNNKDVEIDKDAGNATQQEHVRIIPVQEVTSQTKEDTNTNTNTTTVTS
EDTTNQENTPNDATMEPKTPLSNKEQEKKVRTKEGGNDGKGKRKKKRGRRMGEGYQRYVYRVLKQVHPQMGISSQAMTILNNL
MNDMFEKLADEAAKLTAYTKHMTLTSREIQGAVKLVLPGELGKHAIAEGAKAVTNYVSYVA

>Trifolium pratense - PNY02520.1 histone H3-like protein; **Fabaceae**
MAPKRANKKMVVRSTRKVVEESVQVSVVSSNKRSTRANKDNEIDKDVGSDHDQREEHVRIIPVQEVTPSAKEDSNASTTTFTT
EDKTNQENTPNEATMEPKESENKKVKNKEGNYGKEKRKRKRVRRMGEGYQRYVYRVLKQVHPDMGISFKAMTILNNLMNDMFE
KLADEAAKLTTYIGHMTLSSREIQGAVKLVLPGELGKHAIAEGAKAVTNYISSYGA

Fagales

>Juglans regia - XP_035540958.1 histone H2B.2-like isoform X2; **Juglandaceae**

MAPKRSPKVMGTVMKTRSKVVREIVQVTVVERAQKPTIGEIDEEKEDTFIPSGRKPLRTIAVEDKSEQRNHESAQIPVLVEDK
SSEQENQSFPVQVEGKSKEENQPVQPPVEEKQEEENQDKSPEENQAEVEVPEEEKEKQPTNMTSGKQEGPSKIEGKKKTKSTQ
EGEGKTRRRKRRRKEGGGEEYRRYVYRVLKQVHPELRVSSKAMTVLNNMMKDMFERLTEEAAVLSKYTSRKTLSSREIQGAVR
LVLPGELGRHAIAEGSKAVTSFMSYIARESKRSS

>Morella rubra - KAB1227366.1 Histone H2B type 1-A; **Myricaceae**
MAPKRPPKVVGTVMKTRKFVRETVQVAVVESARKPIPGDVDGDEEGFIQIPEPLRAIVVEDKSEEAKQFVQVQVEEKSEDKKQ
FVQVPVEEKSEEGNQFVRVPAEEKSHEENQPTQAPVEDKSQEGTESVQAPVDDKSPEENQLVQVAEEDRSSQEENLQSDDQVA
EEDKSQEEEKPTTTFKKQEEPSTKEGDKRKARTQEGEGKRRKKRRREGSGEEYRRYVFRVLKQVHPGLEVSSKAMTVLNNMMS
DMFERMADEASKLCKYTGRITLSSREVQGAVKLVLPGELGKHAIAEGSKAVTAYLSHITSSASKS

Malpighiales

>Jatropha curcas - XP_012065091.1 histone H2B.2; **Euphorbiaceae**
MAPKRSGKKKKKVIGTVLRSTRRIIKETVEVSVIEGDPQESTQEYQSQETEEIAANKEPFIRTIPVEEGVHERDDKENEEEAI
EISEEDISLRDQEDFSKKGSASKENLQEQEDLSTKDTTGEENVQEQERPIKKEEKEKTQQEKGQERKKKRKGRGVGREREGYK
RYVFKVLKQVHPELKISSMAMSIINNLMKDMFERIVYEAAKLSDYIHRTTLSSREIQDAVKLVLPGELGRHAIAEGSKAVTNY
MSYKEKRSKA

>Manihot esculenta - XP_021629190.1 histone H2B.4-like; **Euphorbiaceae**
MAPKGKRGKKKVLGTVLRSSKRVIKETVKIAVFEGDTQESTQEDQNGDTEELPENEPLVVRTIPVEERVEEEEEAQTIEVSVK
KPKEEKRKQEKIETHEEKQEPAKTTKTKKRTQEEKGQEKKRRRRRRGVEEGGEGYKRYVFRVLKQVHPELRISSMAMSVINS
LMKDMFERIADEAAKLSQHSHKMTLSSREIQGAVKLVLPGELGRHAIAEGSKAVTNYMSYEAKGSKA

>Populus alba - XP_034905868.1 histone H2B.2-like; **Salicaceae**
MAPKRRGKEVVGTVLRSTKKVVKETVQVAAIENDNQESTQDQDQNGEPEDIDTPALETFRTIPVDEKVHEEEDRVIEVSVEKP
DKEAAIADSQEHIRGPSKEEERQEDQTREVSLRRALKVLIKGDISSGAASRGQQEEPSREDEIRKEDQTGGVSVEEPSKEDPK
EDAASAGDQGKKLGPKKVHPDLGVSSMAMSMINSLMNDMFERIAEEAAKFSDVYRKRTTLSSGEIQGAVKLVLPGELGKHAIA
EGSEAGTNYISHGTKRSKSWRALS

Malvales

>Durio zibethinus - XP_022731946.1 histone H2B.5-like; **Malvaceae**
MAPKLRANVLVRSTEKIVRETVQVAVVQKPEGDSGDQEQLETIPVEDTAEEGERIITEISVEGLSKDKAQKDPQTAQVPGEAP
EDTANTFERTKHLGLQSFEEESSKEAPKALEEERSNECPKASEKEPSNETSKALELEEPPLKKEDERKAKTREGKENNENGKR
KRGKRKELDGNEAYKTYVFRVLKQVHPGMAISSKAMSVINSLMNDMFEKIAEEATKLSKYTERKTLSSREIQGAVRLVLPGEL
GKHAVAEGSKAVTNYASYSEKRPKLD

>Gossypium laxum - MBA0712944.1; **Malvaceae**
MAPKRRAKVVVRSTKKIVKETVQVAVIDKTEGDNNGDQQQLDTVPLEDIEEAEERVITEIPIQGSTEDKAEKEPHKVEAAGEK
NRVQGEEKTEPVHEEEEPRKEEKKGKRKRGKKKELVGHEGYKTYVFRVLKQVHPGMAISSKAMSVINSLMNDMFEKITNEATK
LSQYTDRKTLSSREIQGAVRLVLPGELGKHAVAEGSKAVTNYASYDIKRSKLV

>Herrania umbratica HuH2B8 - XP_021278628.1 histone H2B.2-like; **Malvaceae**
MSKTGETNIKGKRKGRKVTLVKRIAPKRPADVVVRSTKKIVQETVQVAVVQQPEGDSAEQDPMKTVPVEDIAEDEERIITEIS
AERLSKDKAQKEPHTVQVPVEAPGKNKVQKGQEPQQVSAEEPSKDKAQKEPGNEPSIASEDRSKEAPKALEVEDPPLKKEDEK
KEKTGAQEREVKKEEKKGKTRERKESNENRKRKRGKRKEFDGNEAYKTYVFRILKQVHPGMAISSKAMSVINSLMNDMFERIA
DEATKLSKCNERRALSPREIQGAVRLVLPGELGKHAVPEGSKAVTNYASYNGKRPKLN

>Theobroma cacao - XP_007016039.2 histone H2B.2; **Malvaceae**
MAPKRRANVVVRSTKKIVQETVQVAVVQQPEGDRGEQEPMKTVPVEDIAYDEERIITEISVEGLSKDKAQKEPHTVQIPVEAP
GKNKVQKGQEPEQVPTEEPSKDKAQKEPAKEPGNEPSIASEEASEEASKALEVEEPPLKKEAERKEKTGAQEGEEKKDEKNGE
TREGKESNENRKRKRGKRKEFDGNEAYKTYVFRILKQVHPGMAISSKAMSVINSLINGMFERIADEATKLSKYNERRTLSPRE
IQGAVRLVLPGELGKHAVAEGSKAVTNYASYNGKRPKLN

Myrtales

>Eucalyptus grandis - XP_010034182.1 PREDICTED: histone H2B.5-like; **Myrtaceae**
MAPKRKAKVVKTTKKVVQETVEVTLVEKDQNLAAEAENGVETLQQESENEVVKTTVTVEDKLAEEAEQVTVEIPVRQPPQKET
ATEAPRTPRQPEAKDKTQRQKTAQEAKKDRREQEDVEAKNQPKRAREVDEKARGGRRRRRRRVGEGGGEEQYKRYVFRVLKQV
HPGLGISSAAMEVLNGYMNDMFERLAAEAARLSSYAGIKTLSSRDIQGAVRLVLPGELGRHAIAEGAKAVTNYMEHDGGGGGG
AKSKP

>Rhodamnia argentea - XP_030522566.1 histone H2B.2-like; **Myrtaceae**
MAPKRKAKVIKTTRKVVQETVEVALVAKDQKLGAESDEMLQEESESEVVKTTVTVEGELPGGAEQATVEIPIEQPPRQETVTE
APRMPRQPETEAKDKTHDQKTTRDAKEEDREEREEDEETQSPEGTPRAAAEREAETLKEEPALDGERKKAQGTTERERPAERT
EEERSPEKDAEAEKKPKRAREEDEKARVGRRRRRRRVGDGVGAEQYKRYVFRVLKQVHPGLGISSAAMEVLNAYMNDMFERLA
AEAARLSNYAGIKTLTSRDIQGAVRLVLPGELGRHAIAEGAKAVMNYMEHDGGGGGAKSKP

Rosales

>Malus domestica - XP_008359166.3 histone H2B.2-like; **Rosaceae**
MAPKRSAKMVVKTTKRVVKEMVEVSVVKTRRKKQQEDRPLETISVENNDSNQTQNVEVSVGKEPLKTSIIPIETLEQVIPIET
QAENQTLKTQNAEVQVDREAEENPTTPDPQETEKLSKEEEQKSEEDKTLRGGENKDAEDLTKTEEQASKKGEKKSEVKGGKRR
EKRRSRGREEYKTYVYKVLKQVHPGMGVSSKAMTVLNNLMNDMFEKLADEAARLTTYTARKTLSSREIQGAVKLVLTGELGRH
AMAEGTKAVSTYVSYGGGSSKS

>Pyrus ussuriensis - KAB2606623.1 histone H2B.3; **Rosaceae**
MAPKRSAKMVVKTTKQVVKETVEVSVVKTTRKKQQKDQPLKTISIETNESNQNQNVEVSVGKEPVKTSVIPIETQAENQTLKT
QNAEVQVDKEAQENPTTPDPQEAEKSPKEQEQKNEEEKTLGRGENKDAEDLTKTKSKPRRKERRKASRGREEYKIYVYKVLKQ
VHPGMGVSSKAMTVLNNLMYDMFERLADEAARLTTYTARKTLSSREIQGAVRLVLPGELGRHAMAEGTKAVSTYVSYGGGSSK
S

Vitales

>Vitis vinifera - XP_002272312.1 PREDICTED: histone H2B; **Vitaceae**
MAPKRSGKTRSKVVVKATRKVVQQTVEVTVLASKQKPPREEQGKKISKKDKAPEELQREQVSADEEPPKELPTPVTQEEPPKK
EEEKKTTTTQEGREEKKRGRRRRRRTSGRRRKEGGEGYKRYVYRVLKQVHPGLGVSSKAMTVLSGFMNDMFERIAEEAAKLSK
YTGKTTLSAREIQGAVKLVLPGELQKHAMAEGTKAVSNYMDYAAAGGHKQ
